# Antiobesity Effects of Unripe* Rubus coreanus* Miquel and Its Constituents: An* In Vitro* and* In Vivo* Characterization of the Underlying Mechanism

**DOI:** 10.1155/2016/4357656

**Published:** 2016-01-19

**Authors:** Dool-Ri Oh, Yujin Kim, Eun-jin Choi, Hunmi-Lee, Myung-A Jung, Donghyuck Bae, Ara Jo, Young Ran Kim, Sunoh Kim

**Affiliations:** ^1^Jeonnam Bioindustry Foundation, Jeonnam Institute of Natural Resources Research (JINR), Jeollanamdo 529-851, Republic of Korea; ^2^College of Pharmacy, Chonnam National University, Gwangju 500-757, Republic of Korea

## Abstract

*Background*. The objective of the present study was to perform a bioguided fractionation of unripe* Rubus coreanus* Miquel (uRC) and evaluate the lipid accumulation system involvement in its antiobesity activity as well as study the uRC mechanism of action.* Results*. After the fractionation, the BuOH fraction of uRC (uRCB) was the most active fraction, suppressing the differentiation of 3T3-L1 adipocytes in a dose-dependent manner. Moreover, after an oral administration for 8 weeks in HFD-induced obese mice, uRCB (10 and 50 mg/kg/day) produced a significant decrease in body weight, food efficiency ratio, adipose tissue weight and LDL-cholesterol, serum glucose, TC, and TG levels. Similarly, uRCB significantly suppressed the elevated mRNA levels of PPAR*γ* in the adipose tissue* in vivo*. Next, we investigated the antiobesity effects of ellagic acid, erycibelline, 5-hydroxy-2-pyridinemethanol, m-hydroxyphenylglycine, and 4-hydroxycoumarin isolated from uRCB. Without affecting cell viability, five bioactive compounds decreased the lipid accumulation in the 3T3-L1 cells and the mRNA expression levels of key adipogenic genes such as PPAR*γ*, C/EBP*α*, SREBP-1c, ACC, and FAS.* Conclusion*. These results suggest that uRC and its five bioactive compounds may be a useful therapeutic agent for body weight control by downregulating adipogenesis and lipogenesis.

## 1. Introduction

A World Health Organization (WHO) expert committee proposed a classification of overweight and obesity that applies to both men and women and to all adult age groups [1, 2]. Obesity is associated with many metabolic disorders, such as diabetes, gastroesophageal efflux disease, nonalcoholic fatty liver disease, cancer, hypertension, atherosclerosis, and cardiovascular disease [2, 3]. Obesity is associated with excessive food intake and an abnormal accumulation of body fat, defined by a significant increase in body mass index (BMI) [3, 4]. Additionally, obesity arises from an imbalance between energy intake and energy expenditure that eventually leads to the pathological growth of fatty tissue [5]. Obesity-related increases in adipose tissue are the result of a multiplication of fat cells through adipogenesis and increased deposition of cytoplasmic triglycerides [5]. Adipocytes, also known as fat cells or lipocytes, are the cells that primarily compose adipose tissue. They are associated with lipid homeostasis and energy balance [5, 6].

Lipid accumulation reflects the process of preadipocytes differentiating into adipocytes, and this process is regulated by the increased expression of various transcription factors and adipogenesis-related genes, such as peroxisome proliferator-activated receptor *γ* (PPAR*γ*), sterol regulatory element binding protein-1c (SREBP-1c), CCATT/enhancer binding protein *α* (C/EBP*α*), acetyl-CoA carboxylase (ACC), and fatty acid synthase (FAS), which promotes adipogenesis [6]. Increased expression of the transcription factors PPAR*γ* and C/EBP*α* induces adipocyte differentiation by activating the expression of adipocyte-specific proteins such as adiponectin, resistin, and apolipoprotein E, indicating that these transcription factors are important regulators of adipocyte differentiation [7]. At the terminal stage of adipogenesis, fatty acid anabolic genes, such as lipoprotein lipase (LPL) and adipocyte fatty acid-binding protein (aP2), are highly expressed [8, 9].

The fruit of* Rubus coreanus* Miquel, commonly called Bokbunja in Korea, is native to far-eastern Asian countries (South Korea, China, and Japan).* R. coreanus* has been used for centuries as a traditional alternative medicine.* R. coreanus* fruits are usually harvested at the red stage (ripe). When ripe,* R. coreanus* fruits are soft and differ from unripe fruits in color (red versus green, resp.). Various studies have demonstrated that the ripe fruits of* R. coreanus* significantly reduce the risk of many diseases, including asthma, allergies, and obesity [10, 11], and are effective in reducing inflammation and oxidation [12–14]. It has also been reported that* R. coreanus* contains various bioactive compounds, including phenolic acids, triterpenosides, flavonoids, and ellagitannin [15]. A major active compound in* R. coreanus* is ellagic acid, which is in a significantly higher concentration in unripe than ripe* R. coreanus* [16]. Moreover, it has been shown that the ellagic acid possesses antiobesity and antioxidant properties both* in vitro* and* in vivo* [17]. However, the effects of other bioactive compounds of* R. coreanus* on antiobesity have not been investigated. Therefore, further studies will be required in order to define the bioactive compounds that regulate the antiobesity effects of* R. coreanus*.

We previously reported that ripe* R. coreanus* alleviated fatigue by elevating the antioxidative potential [18]. Recently, we reported that the efficacy of the antiobesity effects of unripe* R. coreanus* (uRC) extract was higher than that of the ripe one in high fat diet- (HFD-) induced obese mice [19]. These results might be relevant in determining if the chemical composition changes that occur during ripening are associated with the antiobesity effects of the unripe and ripe fruits. However, the antiobesity effect of the active compounds isolated from uRC remains elusive.

In this context, our aim was to evaluate the involvement of the lipid accumulation system in the antiobesity effect of the active fraction and phytochemically characterize the extract and its active fraction. To evaluate the effects of five organic solvent fractions isolated from uRC on the differentiation of 3T3-L1 adipocytes, we measured lipid accumulation by Oil Red O staining. Moreover, the effects of the BuOH fraction obtained from unripe* R. coreanus* (uRCB) on body weight, adipose tissue weight, serum lipid profiles, and the expression levels of the PPAR*γ* gene associated with lipid metabolism on epididymal adipose tissue in high fat diet- (HFD-) induced obese mice were studied. In addition, to evaluate the effect of bioactive compounds of uRCB on the differentiation of 3T3-L1 adipocytes, we measured lipid accumulation by Oil Red O staining. The inhibitory effects of five candidates, (1) erycibelline, (2) 5-hydroxy-2-pyridinemethanol, (3) m-hydroxyphenylglycine, (4) ellagic acid, and (5) 4-hydroxycoumarin, were further confirmed by downregulation of the expression of PPAR*γ*, C/EBP*α*, SREBP-1c, ACC, and FAS.

## 2. Materials and Methods

### 2.1. Reagents

3-Isobutyl-1-methylxanthine (IBMX), dexamethasone (DEX), insulin, 3-(4,5-dimethylthiazol-2-yl)-2,5-diphenyltetrazolium (MTT), Oil Red O, dimethyl sulfoxide (DMSO), m-hydroxyphenylglycine, p-aminophenol, ellagic acid, and 4-hydroxycoumarin were purchased from Sigma Chemical Co. (St. Louis, MO, USA). Erycibelline was purchased from ChemFaces (Wuhan, China). 5-Hydroxy-2-pyridinemethanol was purchased from IS Chemical Technology (Shanghai, China). Dulbecco's Modified Eagle's Medium (DMEM), bovine serum (BS), and fetal bovine serum (FBS) were purchased from Invitrogen Inc. (Grand Island, NY, USA). All other chemicals were analytical reagent grade.

### 2.2. Preparation of Extract and Its Fractions

The unripe* R. coreanus* (uRC, specimen voucher number: JINR-URC003) fruits used in this study were collected (May 2013) in Gochang County (Jeollabukdo, Republic of Korea) and authenticated by Dr. Kim at the Jeollanamdo Institute of Natural Resources Research (JINR), Jangheung, Jeollanamdo, South Korea. uRC (2.5 kg) was extracted using 20 volumes of 5% ethanol at 100°C for 4 h. The extracted solution was then filtered, concentrated with an evaporator under a vacuum, and freeze-dried. These dried 5% ethanol extracts of uRC were stored at 4°C to avoid compound degradation before they were used in the experiments. The dried 5% ethanol extract of uRC (30 g) was suspended in water and successively divided with *n*-hexane (3 × 500 mL), chloroform (CHCl_3_, 3 × 500 mL), ethyl acetate (EtOAc, 3 × 500 mL), and *n*-butanol (BuOH, 3 × 500 mL).

### 2.3. Cell Culture and Adipocyte Differentiation

3T3-L1 preadipocytes were purchased from the ATCC (American Type Culture Collection, CL-173, Manassas, VA, USA) and cultured in DMEM supplemented with 10% BS. 3T3-L1 cells were seeded at a density of 1 × 10^5^ cells/mL in a six-well plate for 2 days until confluent (designated as day −4). Two days later (designated as day −2), the media were changed to DMEM with 10% BS. Next, two days after confluence (designated as day 0), the cells were cultured in DMEM 10% FBS and a differentiation cocktail (MDI) consisting of 10 *μ*g/mL insulin, 0.5 mM IBMX, and 1 *μ*M DEX, with or without a sample. After 2 days, the cells were cultured in DMEM containing 10% FBS and 10 *μ*g/mL insulin, which was then changed to DMEM containing 10% FBS (designated as day 4). Two days later (designated as day 6), the media were changed to DMEM with 10% FBS. Orlistat (20 *μ*M), and a known antiobesity drug, was used as the positive control [20, 21].

### 2.4. Oil Red O Staining of 3T3-L1 Adipocytes

Oil Red O staining was performed on day 8. The differentiated cells were washed with phosphate buffered saline (PBS) and then fixed in 10% formaldehyde for 30 min. The cells were then washed with distilled water. The fixed cells were stained with an Oil Red O working solution for 30 min and then washed 3 times with distilled water. Differentiation was monitored using microscopy and quantified via elution with 100% isopropanol. Optical density (OD) measurements were obtained at 500 nm.

### 2.5. MTT Assay for the Measurement of Cell Viability

Cell viability was assessed by a MTT assay. 3T3-L1 cells were seeded at a density of 1 × 10^4^ cells/well in a 96-well plate (*n* = 6 replicates) and allowed to adhere overnight. Then, various concentrations of five organic solvent fractions, six subfractions, and bioactive compounds were treated for 24 h. After addition of an MTT solution (5 mg/mL; 50 *μ*L/well) and incubation for 4 h, the supernatants were removed and the formazan crystals were solubilized in 100 *μ*L DMSO. Optical density was determined at 540 nm.

### 2.6. Animals

Male four-week-old C57BL/6 mice weighing 16–21 g were purchased from Central Lab Animal Inc. (Seoul, Republic of Korea). The animals were maintained at a constant room temperature of 22 ± 2°C with a humidity of 50 ± 5% and with free access to water and food under a 12 : 12 h light : dark cycle (lights on at 8:00 am). The animals were acclimatized for 4 days before beginning the experiments. All the experimental procedures were conducted in accordance with the relevant guidelines for the care of experimental animals and were approved by the Jeonnam Institute of Natural Resources Research (approval number JINR-006-2013).

### 2.7. Experimental Groups and Administration

The mice were randomly assigned to four treatment groups consisting of 5 mice each (*n* = 5 per group): normal diet group (ND, *n* = 5), high fat diet group (HFD, *n* = 5), HFD + uRCB 10 mg/kg/day group (uRCB 10, *n* = 5), and HFD + uRCB 50 mg/kg/day group (uRCB 50, *n* = 5). Oral administration of uRCB (10 and 50 mg/kg/day) was continued for 8 weeks from the day of feeding with the HFD. The normal diets consisted of 10% kcal from fat (Research Diets, D12450B, New Brunswick, NJ, USA). The HFD consisted of 60% kcal from fat (Research Diets, D12492, New Brunswick, NJ, USA). Body weights and food intakes were measured twice per week.

### 2.8. Analysis of Biochemical Parameters

At the end of the experimental period, the mice were fasted for 12 h prior to sacrifice. Blood samples were centrifuged at 1,000 g and 4°C for 15 min. Plasma was stored at −70°C prior to the experiments. Serum concentrations of total cholesterol (TC), triglyceride (TG), high-density lipoprotein- (HDL-) cholesterol, low-density lipoprotein- (LDL-) cholesterol, and glucose were measured using an Alere cholesterol LDX system (cholestech LDX, Hayward, CA, USA). Serum glutamic oxaloacetic transaminase (GOT), glutamic pyruvic transaminase (GPT), blood urea nitrogen (BUN), and creatinine levels were measured using the appropriate kits (DRICHEM 4000i, FUGI-FILM, Tokyo, Japan). After the blood collection, the adipose tissue (epididymal fat, perineal fat) and liver were removed from the mice and immediately weighed.

### 2.9. Isolation of Active Subfraction and LC-ESI-MS/MS Analysis

**Figure 1:**
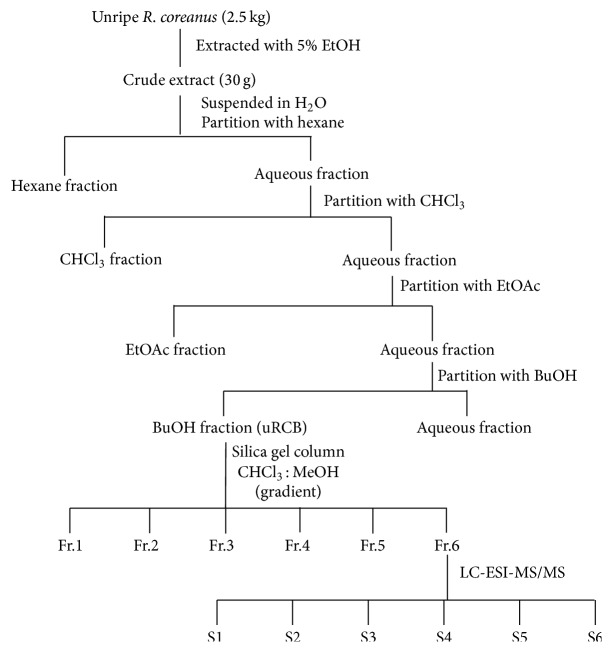
Fractionation procedures for uRC are summarized by a diagrammatic scheme. uRC (30 g) was suspended in water and divided successively with hexane, CHCl_3_, EtOAc, and BuOH. The dried BuOH fraction was eluted on a silica gel column (5 × 40 cm; Merck, 63–200 *μ*m particle size) with a solvent gradient of CHCl_3_ : MeOH (5 : 1 to 0 : 1 ratios) to yield six fractions (Fr.1–Fr.6). Fr.6 was analyzed as six bioactive compounds (S1–S6) using LC-ESI-MS/MS.

The BuOH fraction (2.5 g), which showed a strong lipid accumulation inhibitory effect on 3T3-L1 adipocytes, was chromatographed over a silica gel open column (5 × 40 cm; 63–200 *μ*m particle size, Merck, Darmstadt, Germany) and eluted with a gradient of CHCl_3_/MeOH (5 : 1, 4 : 1, 3 : 1, 2 : 1, 1 : 1, 0 : 1 each 1,000 mL) to yield six subfractions (Fr.1–Fr.6) based on the TLC profile (Figure 1). LC-ESI-MS/MS analysis of the active subfraction (Fr.6) of uRCB was carried out on a 6520 Accurate Q-TOF (Agilent Santa Clara, CA) mass spectrometer coupled to high-performance liquid chromatography (HPLC) equipped with a UV-vis detector. The column used was Agilent Eclipse Plus C18 column (2.1 mm × 150 mm, 1.8 *μ*m). The mobile phase condition consisted of solvent (A) 0.1% formic acid in water and (B) 0.1% formic acid in acetonitrile with a linear gradient elution as follows: starting from 97% solvent A, 0–10 min; 50% A, 10–30 min; 10% A, 30–45 min; and 97% A, 45.1–50 min. The sample wavelength was set at 254 nm. A sample injection volume of 5 *μ*L in methanol and a constant flow rate of 0.3 mL/min were used for the analysis of the sample. The mass spectra were acquired with an ESI source (Agilent Santa Clara, CA). Nitrogen was used as the sheath, and auxiliary gas and helium were used as the collision gas. The ESI MS spectra were acquired in positive ion mode, and a spray voltage of 3.5 kV was employed. The temperature of the heated transfer capillary was 325°C. The mass spectrometer was scanned from *m*/*z* 100 to 1,000 in full scan mode, with a gas flow of 11 L/min, nebulizer 35 psi. Six bioactive compounds were confirmed by a HPLC system (Waters 2998 HPLC systems, USA) with a Capcellpak-C18 column (5 *μ*m, 250 × 4.6 mm; Shiseido, Japan).

### 2.10. RNA Extraction and Reverse Transcription-Polymerase Chain Reaction (RT-PCR)

Total RNA was extracted from the 3T3-L1 adipocytes or epididymal adipose tissue using an easy-BLUE total RNA extraction kit (iNtRON biotechnology, Seongnam, Republic of Korea) according to the manufacturer's instructions. To synthesize cDNA, 1 *μ*g of total RNA was mixed with a premix containing oligo (dT) primer and diethyl pyrocarbonate-treated water in a final volume of 20 *μ*L and incubated at 45°C for 60 min. The reaction was stopped by heat inactivation at 95°C for 5 min. Subsequently, the cDNA was amplified with gene-specific primers using a PCR PreMix (Bioneer, Korea) according to the manufacturer's instructions. The specific primers that we used in this study are shown in Table 1. PCR products were electrophoresed on 1.5% (w/v) agarose gels, stained with ethidium bromide, and photographed. Expression levels were quantified using a gel documentation and analysis system (Gel Doc 2000, Bio-Rad, Sydney, Australia). The relative expression levels of target genes were normalized to a GAPDH internal control.

### 2.11. Statistical Analysis

Data are presented as the mean ± standard deviation (SD), *n* = 6, from three independent experiments with replication. Data were statistically evaluated using Student's *t*-test or one-way analysis of variance (ANOVA) on GraphPad Prism (GraphPad Inc., San Diego, California, USA) software programs. Differences between groups were assessed using Duncan's multiple range tests. Statistical significance was considered at *P* < 0.05.

## 3. Results

### 3.1. Effect of the Organic Solvent Fractions Obtained from uRC on the Intracellular Lipid Accumulation in 3T3-L1 Cells

**Figure 2:**
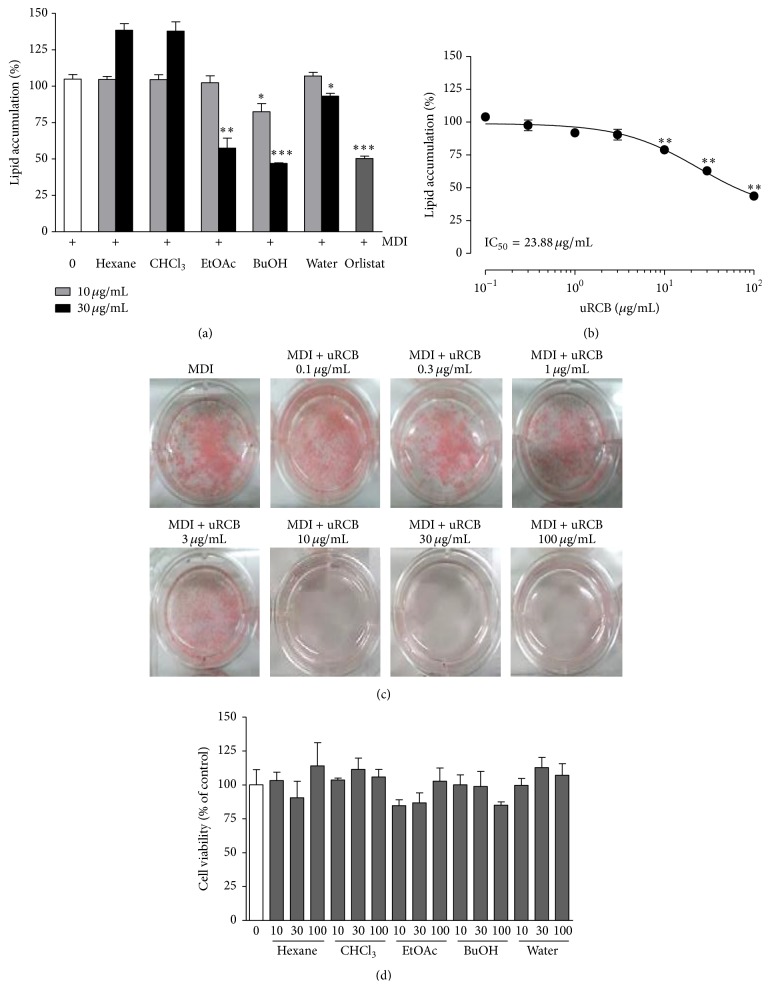
Effect of the organic solvent fractions obtained from uRC on the intracellular lipid accumulation and cell viability in the 3T3-L1 adipocytes. (a) 3T3-L1 cells were treated with organic solvent fractions at concentrations 10 and 30 *μ*g/mL for 8 days. Orlistat (20 *μ*M) was used as the positive control. (b) uRCB were treated at various concentrations (0.1–100 *μ*g/mL) for 8 days. The lipid accumulation in the 3T3-L1 adipocytes was quantified by measuring absorbance. (c) The lipid accumulation was measured using Oil Red O straining. (d) The cell viability was measured using the MTT assay. The values are expressed as the mean ± SD of three experiments. ^*∗*^ *P* < 0.05, ^*∗*^ *P* < 0.01, and ^*∗∗∗*^ *P* < 0.001 compared with the differentiated control.

We previously reported on the antiobesity effects of the 5% ethanol extract of uRC in 3T3-L1 adipocytes and on the body weight, epididymal fat and perirenal fat weights, and lipid profiles in obese mice fed a HFD [19]. Therefore, we screened for the optimal inhibition of lipid accumulation in the organic solvent fractions of the uRC extract during adipocyte differentiation. The 3T3-L1 preadipocytes were treated with various concentrations of organic solvent fractions of uRC for 8 days. The uRC extract was graduated by solvents, such as hexane, CHCl_3_, EtOAc, BuOH, and water (Figure 1). After the incubation, the cells were stained with Oil Red O, and the results are shown in Figure 2. The lipid accumulation inhibitory effects of the EtOAc, BuOH, and water solvent fractions (each 30 *μ*g/mL) were approximately 42.49 ± 3.94% (^*∗∗*^ *P* < 0.01), 53.09 ± 0.25% (^*∗∗∗*^ *P* < 0.001), and 6.79 ± 1.08% (^*∗*^ *P* < 0.05), respectively. Moreover, at low concentrations (10 *μ*g/mL), only the BuOH fraction (17.52 ± 3.20%, ^*∗*^ *P* < 0.05) significantly inhibited the lipid accumulation during adipocyte differentiation. However, there were no significant differences in the lipid level between the concentrations of the hexane and CHCl_3_ fractions. The uRCB at concentrations of 0.1, 0.3, 1, 3, 10, 30, and 100 *μ*g/mL inhibited the lipid accumulation rates in a dose-dependent manner to 0.96 ± 2.83%, 2.43 ± 4.12%, 8.14 ± 2.0%, 9.52 ± 4.08%, 21.05 ± 2.63%, 37.11 ± 2.30%, and 56.28 ± 1.10%, respectively (IC_50_ = 23.88 ± 2.13 *μ*g/mL) (Figure 2(c)). These antiadipogenic effects were achieved at a concentration that did not affect cell viability (*P* > 0.05), according to the MTT assay (Figure 2(d)), and caused no morphological change compared to the control (data not shown). These results indicate that, among the five isolated organic solvent fractions, uRCB effectively blocks adipocyte differentiation in 3T3-L1 preadipocytes.

### 3.2. Effect of uRCB on the Body Weight, Organ Weight, and Serum Biochemical Levels in the HFD-Induced Obese Mice

We investigated the antiobesity effect of uRCB (10 and 50 mg/kg/day, oral administration) in the HFD-induced obese mouse model for 8 weeks. The body weight, food intake, adipose tissue weights, and serum biochemical parameters of each group are shown in Table 2. After 8 weeks, the final body weight gains of the uRCB 10 (31.7 ± 0.2 g, ^*∗∗*^ *P* < 0.01) and uRCB 50 (30.0 ± 0.3 g, ^*∗∗∗*^ *P* < 0.001) mice were significantly lower than that of the HFD mice (40.0 ± 0.6 g). The food intake was not significantly different between the HFD group and the uRCB group. The epididymal fat and perirenal adipose tissue weights in the uRCB 10 (1.67 ± 0.09 g and 0.67 ± 0.03 g) and uRCB 50 (1.48 ± 0.04 g and 0.67 ± 0.01 g) mice were significantly decreased compared with those in the HFD group (2.44 ± 0.04 g and 1.05 ± 0.03 g, resp.) (^*∗∗*^ *P* < 0.01 and ^*∗∗∗*^ *P* < 0.001, resp.). The observed decrease in body weight by uRCB treatment without any significant difference in food intake suggests that uRCB has a physiological effect on the processing of food expenditure. We investigated the effects of uRCB on the serum levels of TC, TG, HDL-cholesterol, and LDL-cholesterol in the HFD-induced obese mouse. uRCB (50 mg/kg) effectively decreased the serum levels of TC, TG, LDL-cholesterol, and glucose compared with the HFD group (^*∗*^ *P* < 0.05 or ^*∗∗*^ *P* < 0.01). In contrast, uRCB significantly increased the levels of HDL-cholesterol in the serum (^*∗*^ *P* < 0.05). Moreover, TC and TG in the serum of groups fed diets containing uRCB (10 mg/kg) were significantly lower compared to those in the HFD group (^*∗*^ *P* < 0.05). Furthermore, uRCB alleviated liver damage by lowering the levels of two common markers for liver damage, GOT, and GPT, which were, in turn, increased by the HFD treatment. The GOT/GPT activities were not increased in the uRCB-administered groups. Instead, they were decreased. However, the relative weights of the livers were not significantly different between the groups. Finally, to evaluate the potential toxic effects of ingesting uRCB, serum toxicological markers indicating kidney injury were measured at the end of the experimental period. The levels of BUN and creatinine were not significantly changed in uRCB-treated mice compared to the levels in the HFD group. These results suggest that the organ and body weight reductions were not caused by liver and kidney toxicity, further suggesting a beneficial role for* Rubus coreanus* extracts against obesity.

### 3.3. Effect of uRCB on PPAR-*γ* Gene Expression in the Epididymal Adipose Tissue of the HFD-Induced Obese Mice

**Figure 3:**
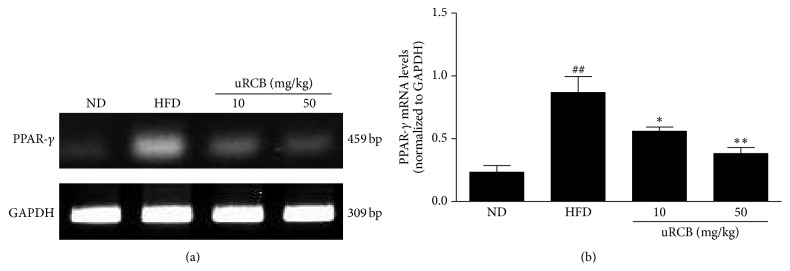
Effect of uRCB on the mRNA levels of peroxisome proliferator-activated receptor *γ* (PPAR*γ*) in the epididymal adipose tissue of the HFD-induced obese mice. The relative mRNA expression levels of the PPAR*γ* gene were normalized against GAPDH. ^##^ *P* < 0.01 compared with the ND group; ^*∗*^ *P* < 0.05, ^*∗∗*^ *P* < 0.01 compared with the HFD group.

PPAR-*γ* is the most important gene during adipogenesis. It plays an important role in adipocyte differentiation, lipid storage, and glucose homeostasis [22, 23]. To confirm the mechanism involved in the uRCB effect on lipid metabolism, the PPAR-*γ* gene expression levels in the epididymal adipose tissue were investigated (Figure 3). Compared with the ND group, the HFD group exhibited increased PPAR-*γ* mRNA levels in the epididymal adipose tissue. However, the PPAR-*γ* mRNA expression levels of uRCB 10 mg/kg (^*∗*^ *P* < 0.05) and uRCB 50 mg/kg (^*∗∗*^ *P* < 0.01) treatment group were significantly decreased compared with those of the HFD group (Figure 3(b)). These results show the suppressive effects of uRCB on adipogenesis by downregulating PPAR-*γ*.

### 3.4. Effect of the Subfractions Obtained from uRCB on the Intracellular Lipid Accumulation in 3T3-L1 Cells

**Figure 4:**
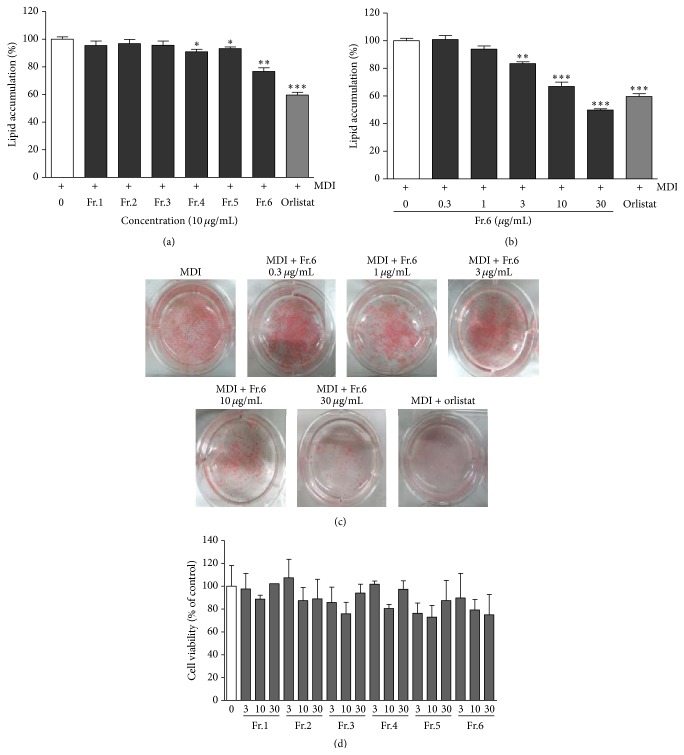
Effect of the subfractions obtained from uRCB on the intracellular lipid accumulation and cell viability in the 3T3-L1 adipocytes. (a) 3T3-L1 cells were treated with subfractions at concentrations (10 *μ*g/mL) for 8 days. (b) The active subfraction (Fr.6) were treated at various concentrations (0.3–30 *μ*g/mL) for 8 days. The lipid accumulation in the 3T3-L1 adipocytes was quantified by measuring absorbance. (c) The lipid accumulation was measured using Oil Red O straining. Orlistat (20 *μ*M) was used as the positive control. (d) The cell viability was measured using the MTT assay. The values are expressed as the mean ± SD of three experiments. ^*∗*^ *P* < 0.05, ^*∗∗*^ *P* < 0.01, and ^*∗∗∗*^ *P* < 0.001 compared with the differentiated control.

uRCB was subjected to column chromatography to yield six subfractions (Fr.1–Fr.6). To screen for the optimal inhibition of lipid accumulation in the subfractions of uRCB during adipocyte differentiation, 3T3-L1 preadipocytes were treated with various concentrations of the subfractions of uRCB for 8 days. After incubation, the cells were stained with Oil Red O. The results are shown in Figure 4. The lipid accumulation inhibitory effects of the subfractions (Fr.1–Fr.6) (each 10 *μ*g/mL) were 4.56 ± 1.89% (not significant), 3.17 ± 1.72% (not significant), 4.39 ± 1.81% (not significant), 9.08 ± 1.04% (^*∗*^ *P* < 0.05), 6.78 ± 0.70% (^*∗*^ *P* < 0.05), and 23.29 ± 1.52% (^*∗∗*^ *P* < 0.01), respectively. Fr.6 showed a strong lipid accumulation inhibitory effect on the 3T3-L1 adipocytes in a dose-dependent manner (Figure 4(b)). The image results were also confirmed by an evaluation of the stain accumulated as lipid droplets (Figure 4(c)). These antiadipogenic effects were achieved at a concentration that did not affect cell viability (*P* > 0.05), according to the MTT assay (Figure 4(d)). These results indicate that, of the six subfractions, Fr.6 effectively blocked adipocyte differentiation in 3T3-L1 preadipocytes.

### 3.5. LC-ESI-MS/MS Analysis of the Active Subfraction 6 (Fr.6) of uRCB

**Figure 5:**
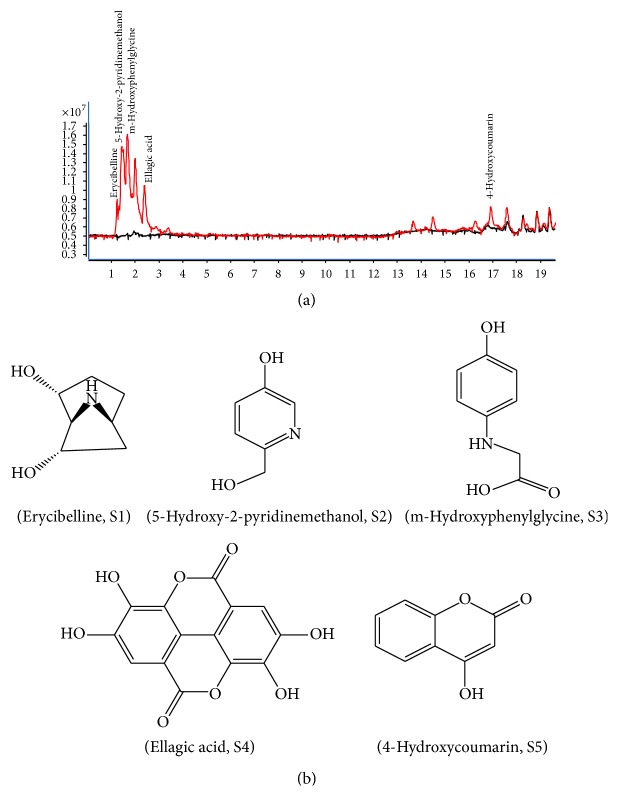
LC-ESI-MS/MS chromatogram and chemical structures of the five bioactive compounds from subfraction 6 (Fr.6). (a) LC-MS/MS analysis: erycibelline, 5-hydroxy-2-pyridinemethanol, m-hydroxyphenylglycine, ellagic acid, and 4-hydroxycoumarin. (b) Structural formulas of the five bioactive compounds.

Five compounds were identified by LC-ESI-MS/MS analysis, and each compound was confirmed by comparing their retention times and UV patterns with reference standard compounds using HPLC analysis. The chromatograms of Fr.6 matched those of erycibelline, 5-hydroxy-2-pyridinemethanol, m-hydroxyphenylglycine, ellagic acid, and 4-hydroxycoumarin (Figure 5).

### 3.6. Cell Viability of Five Bioactive Compounds

**Figure 6:**
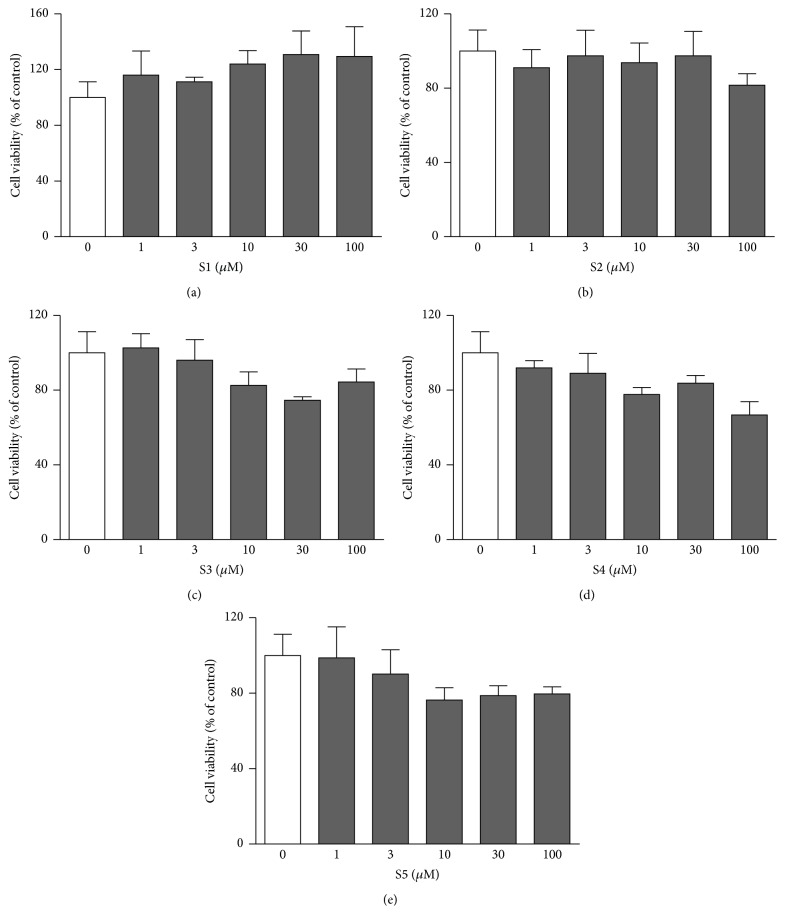
Cell viability in 3T3-L1 adipocytes of the five bioactive compounds. Cell viability was measured using the MTT assay: (a) erycibelline; (b) 5-hydroxy-2-pyridinemethanol; (c) m-hydroxyphenylglycine; (d) ellagic acid; (e) 4-hydroxycoumarin. The values are expressed as the mean ± SD of three experiments. S1: erycibelline, S2: 5-hydroxy-2-pyridinemethanol, S3: m-hydroxyphenylglycine, S4: ellagic acid, and S5: 4-hydroxycoumarin.

To examine the suppressive effects of the six candidates (erycibelline, 5-hydroxy-2-pyridinemethanol, m-hydroxyphenylglycine, ellagic acid, 4-hydroxycoumarin, and p-aminophenol) on 3T3-L1 adipocyte differentiation, we measured the cytotoxicity of various concentrations of the compounds using the MTT assay (Figure 6). The results showed that the viability of the 3T3-L1 cells in the presence of erycibelline, 5-hydroxy-2-pyridinemethanol, m-hydroxyphenylglycine, ellagic acid, and 4-hydroxycoumarin at concentrations of 1, 3, 10, 30, and 100 *μ*M was not decreased (*P* > 0.05). However, p-aminophenol led to a 25.86 ± 0.003% and 21.56 ± 0.002% viability compared to the control at concentrations of 30 and 100 *μ*M, respectively (data not shown). Therefore, it was decided that the noncytotoxic concentrations of up to 100 *μ*M of the five bioactive compounds (erycibelline, 5-hydroxy-2-pyridinemethanol, m-hydroxyphenylglycine, ellagic acid, and 4-hydroxycoumarin) would be used in the following experiments.

### 3.7. Effect of Five Bioactive Compounds on the Intracellular Lipid Accumulation in 3T3-L1 Cells

**Figure 7:**
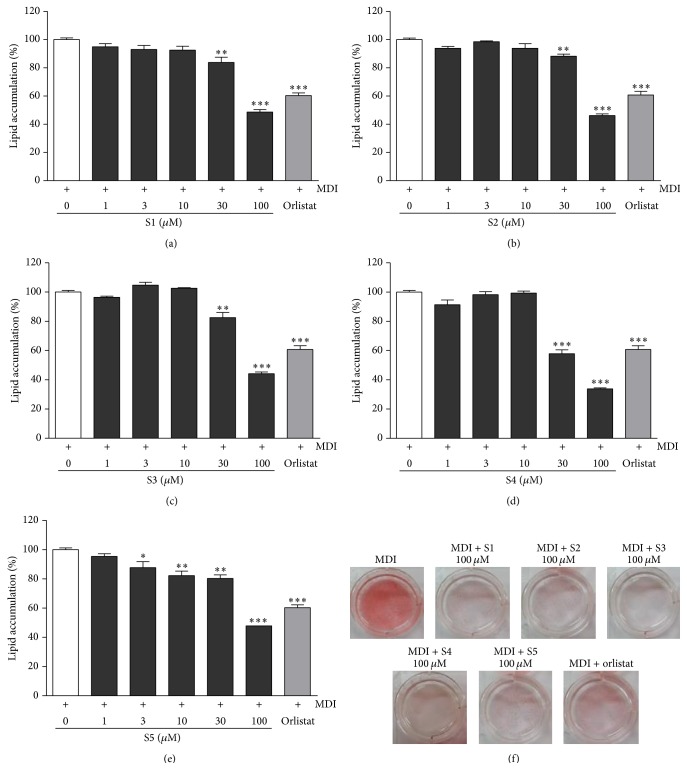
Effect of the five bioactive compounds on lipid accumulation in the 3T3-L1 adipocytes. The lipid accumulation in the 3T3-L1 adipocytes was measured using Oil Red O straining: (a) erycibelline; (b) 5-hydroxy-2-pyridinemethanol; (c) m-hydroxyphenylglycine; (d) ellagic acid; (e) 4-hydroxycoumarin; (f) Oil Red O pictures of the five active compounds. The cells treated with orlistat (20 *μ*M) served as the positive control. The values are expressed as the mean ± SD of three experiments. ^*∗*^ *P* < 0.05, ^*∗∗*^ *P* < 0.01, and ^*∗∗∗*^ *P* < 0.001 compared with the differentiated control. S1: erycibelline, S2: 5-hydroxy-2-pyridinemethanol, S3: m-hydroxyphenylglycine, S4: ellagic acid, and S5: 4-hydroxycoumarin.

To investigate the inhibitory effects of five bioactive compounds (erycibelline, 5-hydroxy-2-pyridinemethanol, m-hydroxyphenylglycine, ellagic acid, and 4-hydroxycoumarin) on adipocyte differentiation, 3T3-L1 cells were treated with various concentrations of the five bioactive compounds for 8 days. After incubation, the lipid accumulation was quantified by Oil Red O staining. The results are shown in Figure 7. The five compounds significantly inhibited lipid accumulation during adipocyte differentiation in a dose-dependent manner. In the cells treated with 100 *μ*M of the five compounds, the lipid accumulation decreased by over 50% compared to the control alone. As shown in Figure 7(f), the five bioactive compounds (erycibelline, 5-hydroxy-2-pyridinemethanol, m-hydroxyphenylglycine, ellagic acid, and 4-hydroxycoumarin) strongly inhibited the differentiation at a concentration of 100 *μ*M. The IC_50_ values of ellagic acid, m-hydroxyphenylglycine, 5-hydroxy-2-pyridinemethanol, 4-hydroxycoumarin, and erycibelline were 52.96 ± 0.60 *μ*M, 89.35 ± 0.88 *μ*M, 93.57 ± 0.94 *μ*M, 95.28 ± 1.57 *μ*M, and 97.25 ± 1.65 *μ*M, respectively.

### 3.8. Suppressive Effects of Five Active Compounds on mRNA Expression of PPAR*γ*, C/EBP*α*, SREBP-1c, FAS, and ACC during 3T3-L1 Differentiation

**Figure 8:**
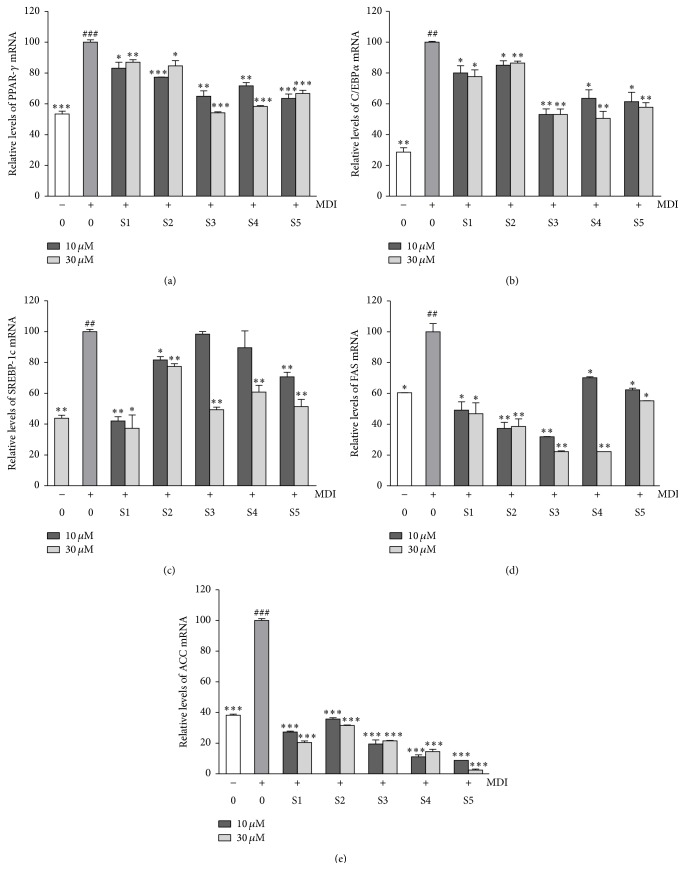
Effect of the five bioactive compounds on the mRNA expression levels of PPAR*γ* (a), C/EBP*α* (b), SREBP-1c (c), FAS (d), and ACC (e). The values are expressed as the mean ± SD of three experiments. ^##^ *P* < 0.01 and ^###^ *P* < 0.001 compared with the control; ^*∗*^ *P* < 0.05, ^*∗∗*^ *P* < 0.01, and ^*∗∗∗*^ *P* < 0.001 compared with the differentiated control. S1: erycibelline, S2: 5-hydroxy-2-pyridinemethanol, S3: m-hydroxyphenylglycine, S4: ellagic acid, and S5: 4-hydroxycoumarin.

To gain a better understanding of the molecular mechanisms underlying the observed antiadipogenic effects, we conducted RT-PCR analysis to examine the influence of the five bioactive compounds on the expression of key transcription factors and lipogenic enzymes (Figure 8). Compared to the differentiated adipocytes in the control cultures, the treatments with the five bioactive compounds significantly decreased the mRNA expressions of PPAR*γ*, C/EBP*α*, and SREBP-1c. We further studied if the five bioactive compounds regulated the expression of the lipogenic enzymes FAS and ACC. A comparison to fully differentiated adipocytes revealed that the treatment with the five bioactive compounds inhibited the expression of FAS mRNA and induced the downregulation of ACC mRNA expression.

## 4. Discussion

Obesity is a major public health problem arising from a chronic positive energy balance from increased food intake, reduced activity, or a combination of both [24, 25]. Obesity leads to serious consequences: type-2 diabetes, hypertension, fatty liver disease, coronary heart disease, stroke, and cancer [26]. Most current obesity treatments, except for surgical removal of the tissue, have failed to result in a sustained reduction of obesity [27]. Drugs used for treating obesity also have serious side effects, including valvular heart disease [28]. Therefore, many studies have recently reported that natural bioactive compounds, such as genistein, quercetin, resveratrol, and epigallocatechin gallate, are effective in treating obesity in 3T3-L1 and mouse models [29–32]. Therefore, in this study, we determine the antiobesity effect of* R. coreanus* and its possible mechanism based on its maturation.* R. coreanus* has been used as a medicine or food depending on its ripeness. uRC has been used as a traditional remedy for some chronic diseases, and ripe* R. coreanus* has been eaten as a fruit.

Adipose tissue is the primary storage organ for excess energy and is also an endocrine organ. Thus, it is important for the regulation of energy homeostasis [33, 34]. An imbalance between the hydrolysis and synthesis of TG is critical during the development of obesity [35]. Lipolysis leads to the breakdown of TG stored in adipose tissue and the release of fatty acids and glycerol [36].

In a previous study, we demonstrated antifatigue effects and antioxidant effects of ripe* R. coreanus* [18]. Recently, we reported that uRC extract had higher efficacy of antiobesity effects compared to ripe RC in HFD-induced obese mice [19]. These results might be relevant in determining if the chemical composition changes that occur during ripening are associated with the antiobesity effects of the unripe and ripe fruits. Our previous another study reported that the contents of malic acid from ripe* Prunus mume* were decreased compared to those from unripe* Prunus mume* [37]. Citric acid, succinic acid, fumaric acid, and ellagic acid were found in unripe and ripe* R. coreanus*; the citric acid and ellagic acid showed the highest concentration in the uRC [16]. Moreover, it has been shown that the citric acid and ellagic acid possess antiobesity and antioxidant properties both* in vitro* and* in vivo* [17]. However, the antiobesity effect of these uRC compounds and the underlying mechanism remain unknown. In the present study, we demonstrated that these fractionized BuOH extracts from uRC and its subfraction 6 strongly suppressed lipid accumulation in 3T3-L1 adipocytes in a dose-dependent manner, without exerting any cytotoxic effects on these cells (Figures 2 and 4). On the basis of these results, we investigated the antiobesity effect of uRCB in a HFD-induced obese mouse model. The final body weight gain of the uRCB-treated mice was significantly lower than that of the HFD-induced obese mice. Moreover, the uRCB treatment group had significantly decreased PPAR-*γ* mRNA expression compared with the mRNA levels in the HFD group.

Woo et al. reported that ellagic acid suppressed 3T3-L1 adipocyte differentiation through a downreduction of the expression of PPAR*γ* and C/EBP*α* mRNA and TG synthetic enzymes [38]. In the present study, we confirmed the inhibitory effects of ellagic acid on lipid accumulation during adipocyte differentiation and investigated the underlying mechanisms by assessing mRNA with RT-PCR. To further our understanding of the effects of the five bioactive compounds (erycibelline, 5-hydroxy-2-pyridinemethanol, m-hydroxyphenylglycine, ellagic acid, and 4-hydroxycoumarin) on lipid metabolism and adipogenesis, we analyzed the mRNA levels of the adipogenic gene and transcription factors that act as master regulators of adipogenesis and lipid storage in 3T3-L1 cells. The differentiation of preadipocytes into adipocytes requires a variety of effectors to activate the process, such as PPAR*γ*, C/EBP*α*, and SREBP-1c. In the present study we showed that the five active compound (erycibelline, 5-hydroxy-2-pyridinemethanol, m-hydroxyphenylglycine, ellagic acid, and 4-hydroxycoumarin) treatments significantly decreased PPAR*γ*, C/EBP*α*, and SREBP-1c mRNA expression compared with the differentiated control cells. Recently, some studies reported that 4-hydroxycoumarin has various biological effects, such as anti-inflammatory [39], antitumor [40], anticoagulant [41], and antibacterial effects [42]. Jung et al. describe that 4-hydroxycoumarin exhibits biological activities as anticoagulant and nonpeptide human immunodeficiency virus (HIV) protease inhibitors [40]. However, the other compounds, such as erycibelline, 5-hydroxy-2-pyridinemethanol, and m-hydroxyphenylglycine, have not been reported as exhibiting any biological effects. The compound p-aminophenol, in contrast, strongly inhibited the differentiation of 3T3-L1 adipocytes (data not shown). However, a cytotoxic effect was observed on the cells treated up to concentrations of 30 *μ*M and 100 *μ*M (data not shown). These results suggest that the five active compounds could play a vital role in the suppression of adipogenesis by downregulating PPAR*γ*, C/EBP*α*, and SREBP-1c. Adipogenesis is the process of cell differentiation by which preadipocytes become adipocytes. PPAR*γ*, a member of the PPAR subfamily of nuclear hormone receptors, and C/EBP*α*, a founding member of a large family of leucine zipper transcription factors, are important regulators of adipogenesis, while SREBP-1c regulates the expression of genes such as FAS and ACC that are involved in lipogenesis [43]. FAS plays an important role in lipogenesis by the regulation of fatty acid synthesis. ACC is another key enzyme in lipogenesis that is regulated by the transcription factor SREBP-1c. Given that the inhibition of ACC is a crucial step in the reduction of lipid accumulation, this enzyme and FAS should be targeted in antiobesity research. In the present study, we showed that the five active compound treatments significantly decreased FAS and ACC mRNA expression compared with that in differentiated control cells. Taken together, these results demonstrate that the inhibition of lipogenesis in differentiated 3T3-L1 adipocytes by treatment with uRC may be mediated in part through downregulation of the adipogenic transcription factors PPAR*γ*, C/EBP*α*, and SREBP-1c and the target genes FAS and ACC. Although the five active compounds are the components that have antiobesity properties in* R. coreanus*, further studies will be required in order to define the functional compounds that regulate the antiobesity effects of uRC.

## 5. Conclusion

In conclusion, this study is the first to report that uRCB and its five active compounds (erycibelline, 5-hydroxy-2-pyridinemethanol, m-hydroxyphenylglycine, ellagic acid, and 4-hydroxycoumarin) inhibit adipocyte differentiation through the suppression of transcriptional factors, including PPAR*γ*, C/EBP*α*, and SREBP-1c, the adipogenesis related gene (ACC), and enzymes (FAS). In addition, uRCB decreased the body weight, adipose tissue weight (epididymal and perirenal fat pad), and serum TC/TG, glucose, and LDL-cholesterol levels in HFD-induced obese mice. Taken together, uRC, which has been used as a traditional remedy, might be used as a potential functional material for the prevention and treatment of obesity because of its antiadipogenic effects. An animal study is under way to evaluate the antiobesity effects of active compounds using a HFD-induced obese mouse model.

## Figures and Tables

**Table 1:** Primers and expected sizes of PCR products with each primer pair.

Gene	Primer sequence (5′→3′)	Product size (bp)	GenBank accession number
PPAR*γ*	Forward: GACCACTCGCATTCCTTT	459	NM_001127330.1
	Reverse: GGCATTGTGAGACATCCC		

C/EBP*α*	Forward: CTATCAGTCTGTCCAGCCC	302	NM_007678.3
	Reverse: CTATCAGTCTGTCCAGCCC		

SREBP-1c	Forward: AAGGGCCAGGAGTGGGTAAAC	226	NM_011480.3
	Reverse: CGCGCCGCGCCCCATTAGG		

FAS	Forward: CAGTTCCAGCCATGAAGAGA	192	NM_007988.3
	Reverse: TTTGCTGGCAAAGAGAACAC		

ACC	Forward: TCTATTCGGGGTGACTTTC	350	NM_133360.2
	Reverse: CTATCAGTCTGTCCAGCCC		

GAPDH	Forward: AGATCCACAACGGATACATT	309	NM_008084
	Reverse: TCCCTCAAGATTGTCAGCAA		

PPAR*γ*: peroxisome proliferator-activated receptor *γ*, C/EBP*α*: CCAAT/enhancer binding protein *α*, SREBP-1c: sterol regulatory element binding protein 1c, FAS: fatty acid synthase, ACC: acetyl-coenzyme A, and GAPDH: glyceraldehyde-3-phosphate dehydrogenase.

**Table 2:** The effects of uRCB on body weight, food intake, adipose tissue weight, and serum lipid parameters in HFD-induced obese mice.

	ND	HFD	uRCB 10 mg/kg	uRCB 50 mg/kg
Initial body weight (g)	20.64 ± 0.08	20.92 ± 0.09	20.89 ± 0.31	20.78 ± 0.23
Final body weight (g)	26.63 ± 0.49	40.05 ± 0.65^###^	31.67 ± 0.24^*∗∗*^	30.05 ± 0.25^*∗∗∗*^
Body weight gain (g)	5.99 ± 0.47	19.13 ± 0.71^###^	10.78 ± 0.42^*∗∗*^	8.90 ± 0.43^*∗∗∗*^
Food intake (g/day)	2.75 ± 0.04	2.16 ± 0.11	2.48 ± 0.00	2.68 ± 0.09
FER^(1)^	2.22 ± 0.17	7.96 ± 0.32^###^	4.31 ± 0.17^*∗∗∗*^	4.27 ± 0.41^*∗∗∗*^
Organ weight				
Epididymal fat (g)	0.49 ± 0.01	2.44 ± 0.04^###^	1.67 ± 0.09^*∗∗*^	1.48 ± 0.04^*∗∗∗*^
Perirenal fat (g)	0.16 ± 0.01	1.05 ± 0.03^###^	0.67 ± 0.03^*∗∗*^	0.67 ± 0.01^*∗∗∗*^
Liver (g)	1.29 ± 0.04	1.51 ± 0.07	1.19 ± 0.02	1.14 ± 0.02
Blood parameter				
GOT (U/L)	95.20 ± 2.57	182.40 ± 14.85^#^	103.83 ± 2.33^*∗*^	90.60 ± 1.61^*∗*^
GPT (U/L)	29.2 ± 3.31	42.80 ± 2.47	38.17 ± 1.76	29.80 ± 0.75
Creatinine (mg/dL)	0.32 ± 0.02	0.16 ± 0.01	0.15 ± 0.01	0.10 ± 0.00
BUN (mg/dL)	27.10 ± 1.04	27.88 ± 0.87	26.65 ± 0.57	27.28 ± 0.37
Glucose (mg/dL)	187.20 ± 5.48	300.50 ± 12.52^#^	224.67 ± 4.32	182.25 ± 5.32^*∗*^
TC (mg/dL)	124.20 ± 1.73	195.20 ± 3.11^###^	181.67 ± 3.37^*∗*^	171.50 ± 2.27^*∗*^
TG (mg/dL)	173.00 ± 6.14	247.20 ± 10.84^#^	188.67 ± 5.02^*∗*^	186.80 ± 5.98^*∗∗*^
HDL-C (mg/dL)	76.67 ± 1.19	57.00 ± 1.10^#^	61.67 ± 2.96	75.67 ± 2.03^*∗*^
LDL-C^(2)^ (mg/dL)	22.13 ± 2.42	83.33 ± 2.68^###^	80.40 ± 3.90	72.13 ± 3.39^*∗*^

^(1)^FER (food efficiency ratio) = body weight gain/food intake

^(2)^LDL-C (LDL-cholesterol) = total cholesterol − HDL-cholesterol − triglyceride/5

Values are expressed as mean ± SE (*n* = 5). ^#^ *P* < 0.05 and ^###^ *P* < 0.001 compared with the ND group; ^*∗*^ *P* < 0.05, ^*∗∗*^ *P* < 0.01, and ^*∗∗∗*^ *P* < 0.001 compared with the HFD group.
